# Guest Edited Collection: Radioisotopes and radiochemistry in health science

**DOI:** 10.1038/s41598-019-56278-1

**Published:** 2020-01-10

**Authors:** Michael E. Fassbender

**Affiliations:** Chemistry Division, Los Alamos National Laboratory, Los Alamos, NM 87545 United States

**Keywords:** Nuclear chemistry, Analytical chemistry

## Abstract

Radioisotopes can be produced artificially from stable nuclei through the interaction with particles or highly energetic photons. In combination with modern detection and counting techniques, radioisotopes and radiochemical methods uniquely contribute to the health sciences. This Collection showcases salient aspects of medical radioisotope science ranging from the production, recovery and purification of radioisotopes to the methods used to attach them to biomolecules. The Collection also presents studies that highlight the importance of radiochemistry in the assessment of environmental radioactivity.

## Introduction

The discovery of radioactivity dates back to the year 1896, when Antoine Henri Becquerel, now the eponym of the SI unit to quantify radioactive decay rate, first described the phenomenon of a mysterious penetrating radiation originating from uranium salt^1^. This radiation was able to produce an image on a photographic plate. Radioactivity is a process where an unstable nucleus undergoes conversion to a different -energetically more favorable- nucleus, accompanied by the release of energy in the form of particles and photons.

The relationship between radioactivity and human health perception has been a bumpy ride. The radioactive element radium (^226^Ra half-life 1600 y) that was discovered in the year 1898^2^ by Polish-French radiochemist Marie Sklodowska-Curie, the graduate student of Becquerel who would later earn herself the honour of becoming the first female Nobel prize laureate, was initially regarded as a harmless, even health-boosting natural agent. A few milligrams of radium chloride ^226^RaCl_2_ could be recovered from one metric ton of uranium ore^3^ through tedious radiochemical efforts. Grossly underestimating the hazards, radium was made a component of self-luminous paints^4^; it was also the subject of flourishing radioactive quackery^5^.

A later discovery, for which German radiochemist Otto Hahn received the Nobel Prize in the year 1944, ushered civilization into the “nuclear age”. Hahn –initially unknowingly- provided the radiochemical proof of barium as a product of neutron bombardment of uranium^6^, which signalled the existence of a thitherto-unknown phenomenon: nuclear fission. Nuclear fission could be harnessed as a source of energy, but the wide range of unstable fission products associate it with quasi everlasting environmental impact -highly geochemically mobile^7^ ^99^Tc (half-life 2.1·10^5^ y) to mention here- and dangerous levels of radioactivity.

Decades later there was the “Chernobyl disaster”^8,9^. Still a high schooler in Cold-War era West Germany, I vividly recall the news streaming in that stoked the populace’s fears of radioactivity like no other event before. And the event on April 26^th^, 1986, made us Europeans think twice about what produce to eat and to better ask where it came from. Children were barred from entering playgrounds; the time span “eight days”, the half-life of volatile fission product ^131^I, kept circulating in people’s heads. The “Chernobyl” and “Fukushima”^10^ disasters are certainly entries in the history annals of nuclear science and technology that bear witness to the blight of radioactivity.

The blessings of radioactivity, on the other hand, became apparent from the work of another radiochemist and Nobel Prize laureate, George de Hevesy. Hevesy pioneered the concept of radioactive tracers to study biological processes *in vivo*^11^, and he is widely regarded as the “father of modern nuclear medicine”^12^. Other sources^13^ name the Donner Laboratory at the University of California, Berkley, the “birthplace of nuclear medicine” and rather ascribe nuclear fatherhood to John Lawrence. The discovery of several well-known medical isotopes was claimed by the Donner Laboratory, including ^14^C, ^18^F, ^15^O and ^201^Tl. Also among them was “an isotope with a half-life of about one week”^13^, as personally requested by Joseph G. Hamilton from Glenn T. Seaborg (1951 Chemistry Nobelist) for use in certain thyroid studies. Iodine-131 (half-life 8.0 d) was Seaborg’s legendary answer to his request, produced at Berkeley via deuteron irradiation of tellurium. The first use of ^131^I to treat hypothyroidism in humans was reported by Saul Hertz on January 1^st^, 1941^14^, and a series of treatment studies was published in May 1946^15^. Ironically, the later “Chernobyl scare” ^131^I had become the first U.S. Food and Drug Administration approved radiopharmaceutical in 1951^16^. Seaborg –along with Emilio Segré - also isolated metastable ^99m^Tc (half-life 6.0 h) for the first time^17^. This isotope has become an invaluable tool for single photon computer tomography (SPECT). The last radioisotopes to mention in my almost chiastic introductory narrative are alpha emitters ^223^Ra and ^224^Ra^18^ (half-lives 11.4 d and 3.7 d), thereby taking the reader’s attention back to the element that Madame Curie discovered. One of them, ^223^Ra, has recently become a U.S. FDA approved pharmaceutical for the treatment of bone metastases.

## Collection Overview

As the ‘Radioisotopes and radiochemistry in health science’ Collection launches, applications of short-lived positron emitter fluorine-18 (half-life 109.8 min) naturally find the strongest representation. Certainly due to fluorine’s ability to rapidly form extremely stable F-C bonds via nucleophilic or electrophilic fluorination of organic molecules, ^18^F is undisputedly the most widely used positron emission tomography (PET) labelling agent. Advances in ^18^F labelling chemistry also shed light on the more fundamental chemistry of this most reactive halogen. For instance, a new route to [^18^F]fluoroform has been demonstrated^19^, where the trifluoromethyl synthon is formed in the gas phase by passing [^18^F]fluoromethane over heated CoF_3_, one of the few binary phases that release elemental fluorine (F_2_) upon heating. The introduction of new ^18^F-labelled triazolyl-linked argininamides to target neuropeptides for the imaging of mammary carcinoma^20^ serves as an apt example of click chemistry-based ^18^F-labelling, and a Design of Experiments (DoE) approach has been reported to optimize copper-mediated radiofluorination^21^. The evaluation of new ^18^F-labelled melanoma xenograft targeting peptides is the subject of an imaging study^22^ that is also published as part of this Collection.

While the lightest halogen has become the workhorse of PET imaging, the heaviest (natural) halogen, astatine, has been advancing as a therapy agent. This Collection includes reports on optimized ^209^Bi(α,2n) cyclotron production and recovery of targeted alpha therapy (TAT) isotope ^211^At (7.2 h)^23,24^ and an investigation into astatine’s solvent extraction behaviour^25^. Radioiodine still plays an important role; beyond historic ^131^I, other iodine isotopes^26^ are now utilized for SPECT imaging (^123^I, 13.3 h) and treatment (Auger emitter ^125^I, 59.4 d).

Radiometals have their deserved place in the toolbox of nuclear medicine: cobalt (^57^Co, 271.8 d; as a surrogate for ^55^Co, 17.5 h) and indium (^111^In, 2.8 d) were used as labels for affibody monomers targeting tumours in BxPC-3 xenografted mice^27^. A further contribution broaches the application of nanospheres labelled with ^89^Zr (78.4 h)^28^, a positron emitter with a half-life roughly matching the *in vivo* circulation of antibodies.

Lanthanides are represented, as many of their easily accessible chemically similar -and thus interchangeable- radioisotopes constitute versatile agents for diagnostics and therapy. The purification of SPECT isotope ^155^Tb (5.3 d) is featured^29^, and a method for the production and recovery of the theranostic pair ^132,135^La (4.8 h and 19.5 h) with initial imaging and biodistribution evaluation is reported^30^. Alpha emitter ^225^Ac (10.0 d) has emerged as a TAT isotope: one contribution looks into the *in-vivo* redistribution of ^225^Ac daughter isotopes in a mouse model^31^; another study reports the purification of accelerator produced ^225^Ac using a silicotitanate sorbent^32^.

Surveys into the distribution and detection of naturally occurring radioactivity are an important part of the Collection as well, as they demonstrate how radioisotope science helps assess radiation doses from natural radioactivity; the “isodose” concept to Swedish residential buildings was applied to optimize topsoil removal concepts^33^.

As the Collection is still open for submissions on a rolling basis, this is only the beginning! May the interested colleague find in this “living” Collection a one-stop overview of the current research that puts radioactivity and radiochemistry to work for the sake of human health.
